# Protein Family Expansions and Biological Complexity

**DOI:** 10.1371/journal.pcbi.0020048

**Published:** 2006-05-26

**Authors:** Christine Vogel, Cyrus Chothia

**Affiliations:** 1 Medical Research Council Laboratory of Molecular Biology, Cambridge, United Kingdom; 2 Institute for Cellular and Molecular Biology, University of Texas at Austin, Austin, Texas, United States of America; University of California San Diego, United States of America

## Abstract

During the course of evolution, new proteins are produced very largely as the result of gene duplication, divergence and, in many cases, combination. This means that proteins or protein domains belong to families or, in cases where their relationships can only be recognised on the basis of structure, superfamilies whose members descended from a common ancestor. The size of superfamilies can vary greatly. Also, during the course of evolution organisms of increasing complexity have arisen. In this paper we determine the identity of those superfamilies whose relative sizes in different organisms are highly correlated to the complexity of the organisms. As a measure of the complexity of 38 uni- and multicellular eukaryotes we took the number of different cell types of which they are composed. Of 1,219 superfamilies, there are 194 whose sizes in the 38 organisms are strongly correlated with the number of cell types in the organisms. We give outline descriptions of these superfamilies. Half are involved in extracellular processes or regulation and smaller proportions in other types of activity. Half of all superfamilies have no significant correlation with complexity. We also determined whether the expansions of large superfamilies correlate with each other. We found three large clusters of correlated expansions: one involves expansions in both vertebrates and plants, one just in vertebrates, and one just in plants. Our work identifies important protein families and provides one explanation of the discrepancy between the total number of genes and the apparent physiological complexity of eukaryotic organisms.

## Synopsis

One of the main goals in biology is to understand how complex organisms have evolved. Much of an organism's physiology, and hence complexity, is determined by its protein repertoire. The repertoire has been largely formed by the duplication, divergence, and combination of genes. This means that proteins can be grouped into families whose members are descended from a common ancestor. The authors have examined the sizes of 1,219 protein families in 38 eukaryotes of different complexity. Only a small fraction of protein families have expansions that are correlated with the number of cell types in the organisms. Half of these families are involved in regulation or extracellular processes. Other families do have expansions but in a lineage-specific manner. Thus, certain protein family expansions are “progressive” in that they lead to increases in biological complexity; other expansions are “conservative” in that they help an organism to adapt better to its environment, but do not increase its complexity. This means that there is no simple correlation between an organism's complexity and the number of its genes.

## Introduction

During the course of evolution, the complexity of organisms as measured by the total number of their cells and the number of different cell types has increased greatly. The different processes that have produced these increases in biological complexity are of fundamental interest, and the data available from complete genome sequences should allow us to eventually determine their general nature and relative contributions. Prior to the information available from the genome projects, it was believed that one central process is the formation of new genes by gene duplication, divergence, and combination [1–6]. Particular emphasis was placed on extensions in the repertoire of proteins involved in the regulation of expression and in signal transduction; for a review see Kirschner and Gerhart [7].

From analyses of prokaryote genome sequences, van Nimwegen [8] and Ranea et al. [9] have shown that the number of genes in different functional categories scales as a power-law of the total number of genes. For different functional categories, the exponent of the power-law has different values. High values, ~2, are indeed found for proteins involved in transcription and its regulation and for those involved in signal transduction. Low values, <0.5, are found for those involved in protein biosynthesis, the cell cycle, and DNA replication [8]. Other functional groups have intermediate values. Van Nimwegen also obtained somewhat similar results from an analysis of the eukaryote genome sequences available at the time he carried out that work [8].

In eukaryotes, a comparison of the predicted protein sequences of the unicellular yeast, *Saccharomyces cerevisiae,* and the multicellular nematode, *Caenorhabditis elegans,* also showed that the nematode has many more proteins, and types of proteins involved in transcription and its regulation, and in signal transduction, than yeast [10]. Subsequently, an analysis of the proteins with these functions in the plant [11], the fly Drosophila melanogaster [12], and in humans [13] showed that repertoire of these proteins becomes larger and more diverse as complexity increases.

**Figure 1:**
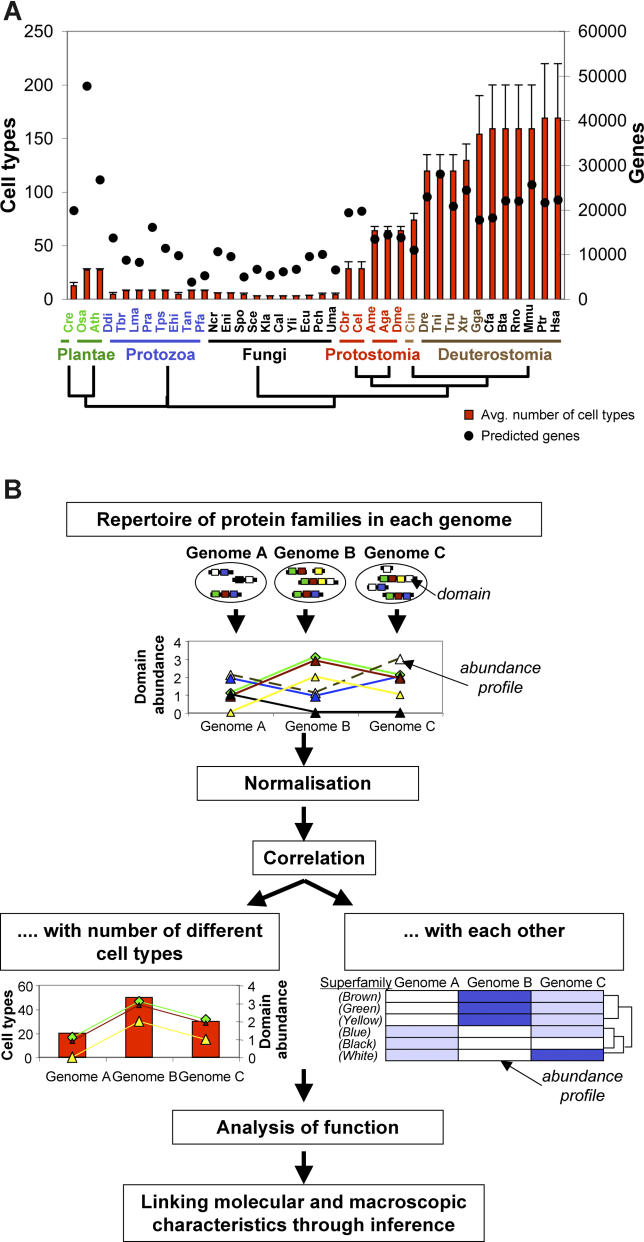
Motivation and Outline of the Analysis (A) The number of genes and eukaryotic complexity are uncorrelated. The figure displays for 38 eukaryotic genomes the estimated number of different cell types [28,29] in relation to the predicted total number of genes. The tree indicates, in a simplified form, the phylogenetic relationships between the organisms as taken from the National Center of Biotechnology Information (NCBI) taxonomy server (http://www.ncbi.nlm.nih.gov/Taxonomy). The order of the organisms is the same in all figures and tables; their major groups are: plants (green), protozoa (blue), fungi (black), and animals (red and brown). The correlation between the number of different cell types and the number of genes is poor (*R^2^* = 0.29, *R* = 0.54). Within the plants, we distinguish green algae *(Cre, Chlamydomonas reinhardtii),* and flowering plants *(Osa, O. sativa; Ath, Arabidopsis thaliana).* We include eight protozoa *(Ddi, Dictyostelium discoideum; Tbr, Trypanosoma brucei; Lma, Leishmania major; Pra, Phytophthora ramorum; Tps, Thalassiosira pseudonana; Ehi, Entamoeba histolytica; Tan, Theileria annulata; Pfa, Plasmodium falciparum),* and ten fungi *(Ncr, Neurospora crassa; Eni, Emericella nidulans; Spo, Schizosaccharomyces pombe; Sce, S. cerevisiae; Kla, Kluyveromyces lactis; Cal, Candida albicans; Yli, Yarrowia lipolytica; Ecu, Encephalitozoon cuniculi; Pch, Phanerochaete chrysosporium; Uma, Ustilago maydis).* Protostomia include two nematodes *(Cbr, Caenorhabditis briggsae; Cel, C. elegans),* and three insects *(Ame, Apis mellifera; Aga, Anopheles gambiae; Dme, D. melanogaster).* Deuterostomia include one urochordate *(Cin, Ciona intestinalis),* and 11 vertebrates, among which six are mammals *(Dre, Danio rerio; Tni, Tetraodon nigroviridis; Tru, Takifugu rubripes; Xtr, Xenopus tropicalis; Gga, Gallus gallus;* and *Cfa, Canis familiaris; Bta, Bos taurus; Rno, Rattus norvegicus; Mmu, Mus musculus; Ptr, Pan troglodytes;* and *Hsa, H. sapiens,* respectively). (B) Outline of our analysis. For each of the 38 genomes (three, symbolised by circles), we collected information on the number of proteins (lines with boxes) that contain domains of particular superfamilies (boxes of particular colour). The resulting abundance profiles were normalised and compared both to the estimated number of different cell types in each organism, and to each other. Analysis of function of particular groups of domain superfamilies gives information on how their expansion in some organisms may have supported an increase in organismal complexity.

However, in eukaryotes there is a complication not found in prokaryotes: the biological complexity of an organism is not correlated with its total number of genes (Figure 1A, *R* = 0.54). The current known number of genes are 26,750 in the plant *Arabidopsis,* 20,050 in the nematode *C. elegans,* 13,770 in *Drosophila,* and 22,220 in the vertebrate Homo sapiens [14] (Figure 1A). This implies that, whilst the expansion of some protein families can lead to the evolution of organisms of higher complexity, other protein families expanded to improve an organism's adaptation to its environment but without a substantial change in complexity. These two types of expansion have been called “progressive” and “conservative” protein family expansions, respectively [15].

In this paper, we determine the extent to which the expansion of individual protein families and combinations of families correlates with increases in macroscopic complexity of organisms such as fungi, protozoa, plants, nematodes, insects, a urochordate, and vertebrates. We measure protein family expansions in terms of the number of proteins that contain domains of defined superfamilies [16] (Figure 1B and Dataset S1). Domains are the structural, functional, and evolutionary units that form proteins; and domains of common ancestry are grouped into superfamilies based on evidence from structure, sequence, and function [16]. Two proteins that contain a domain of the same superfamily are grouped into one protein family.

We carry out two sets of calculations. First, we determine whether families undergo different expansions in different genomes and whether or not the expansions are related to the complexity of the organisms from which they come. As a measure of biological complexity of an organism, we use its number of different cell types. Second, we determine the extent to which the abundance of different superfamilies within different sets of the genomes correlate with each other. We identify three major trends that encompass about half of the largest superfamilies.

## Results/Discussion

The 38 eukaryote genomes used in this work comprise those from 11 vertebrates, among which five are mammals from one urochordate, five protostomia, including two nematodes and three insects, from ten fungi, eight protozoa, and from three plants, of which two are flowering (magnoliophyta). The phylogenetic relationships of these organisms are indicated in Figure 1A. We focus on the relationships between organisms and their protein repertoires at the level of major phylogenetic groups, such as uni- and multicellular plants, protozoa, fungi, protostomia, the urochordate, vertebrates, and, within the latter ones, mammals, but do not resolve relationships within these groups. Further, our analysis is limited to those 60% of proteins for which there is good information on the evolutionary relationships of the domains of which they are composed, as well as information on domain functions. Before turning to our results, we describe briefly how the repertoire of domain superfamilies is defined and predicted in genome sequences, and which types of functions they usually have.

### Domain Superfamilies as Units of Protein Evolution

Our ability to detect the evolutionary relationships of proteins, or protein domains, by sequence comparisons is limited because they frequently diverge beyond the point where their true relationship can be recognised by such comparisons. Also, large proteins are formed by combinations of domains that often come from different superfamilies. The presence or absence of superfamily relationships and of different domains can be determined if the three-dimensional structure of proteins is known, and these relationships are described in the Structural Classification of Proteins database (SCOP) [16]. It is these proteins and domains of known structure and their clear homologues for which we searched in the genome sequences of the 38 organisms.

The SUPERFAMILY database [17] contains hidden Markov models of the one-domain proteins and of the individual domains in multidomain proteins that are in the SCOP database. The SUPERFAMILY database also contains a description of the significant matches that the hidden Markov models make to the protein sequences predicted to the known genomes. Matches are made to all or part of about 60% of the predicted proteins in each genome. We extracted from SUPERFAMILY the matches made by the hidden Markov models to the sequences of 38 eukaryotes and placed them in their respective superfamilies. This procedure resulted in 1,219 domain superfamilies that occur in at least one protein in at least one of the 38 genomes. In human, for example, we find 950 of these superfamilies, and they map to a total of 19,225 domains [18]. In our analysis, we sometimes refer to a subset of largest superfamilies; these are the 299 superfamilies that occur in at least 25 proteins in at least one of the genomes.

### The Functions of Superfamily Members

In an extension of domain annotations described previously [19], we manually assigned each superfamily to one of 50 types of function from a scheme similar to that used in COGs (clusters of orthologous groups of proteins) [20]. The annotation is based on information taken from SCOP [21], Pfam [22], SwissProt [23], and literature. Each of these functions map to one of seven general categories (see Protocol S1 and http://polaris.icmb.utexas.edu/people/cvogel/HV ): (1) Information: storage and maintenance of the genetic code, DNA replication/repair, general transcription/translation; (2) Regulation: regulation of gene expression and protein activity, information processing in response to environmental input, signal transduction, general regulatory or receptor activity; (3) Metabolism: anabolic and catabolic processes, cell maintenance/homeostasis, secondary metabolism; (4) Intracellular processes; cell motility/division, cell death, intracellular transport, secretion; (5) Extracellular processes: inter- and extracellular processes (e.g., cell adhesion), organismal processes (e.g., blood clotting), immune system; (6) General: general and multiple functions, interactions with proteins/ions/lipids/small molecules; and (7) Other/Unknown: unknown function, viral proteins/toxins.

We are aware that the members of some superfamilies, particularly the large ones, may have a variety of functions. For example, immunoglobulin domains are involved in cell adhesion, muscle structure, the extracellular matrix, and the immune system. The function categories here aim to describe the dominant and most widespread function for each superfamily, as far as it is known today. We annotated all 1,219 domain superfamilies of seven SCOP classes *a* to *g* [21] that occur in the 38 genomes. Close to half of all superfamilies (448) have metabolism-related functions, while each of the other categories comprises less than 15% of the domain superfamilies (Table 1). In humans, one-third of the superfamilies are metabolic (339/950), mapping to one-sixth of all domains (3,212/19,225). Some 10% of the superfamilies (122) have unknown functions (also see Figure S1).

**Table 1:**
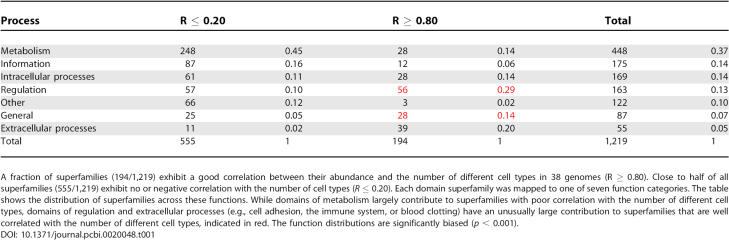
Few Domain Superfamilies Correlate Well with the Number of Different Cell Types

### Family Expansions and the Number of Different Cell Types

We aim to identify superfamilies whose expansions may have supported an increase in biological complexity in some eukaryotes as compared to others, thereby linking molecular characteristics to a macroscopic phenotype. As a measure of the biological complexity of an organism, we would ideally use information on both the number of cell types and the total number of cells. While some previous work is available for closely related organisms on correlates of their body mass [24–27], information on the total number of cells is not readily available for a wide range of organisms such as those used in our analysis. Therefore, we use here as a measure of complexity the estimates made for the number of different cell types found in different organisms [28,29]. Fungi and protozoa have the lowest complexity with five or fewer cell types; vascular plants have a similar number of different cell types as nematodes (i.e., ~30 or fewer), and vertebrates are of highest complexity with some 170 different cell types (Figure 1 and Dataset S1).

For each individual domain superfamily, we calculated the correlation between its abundance profile and the estimated number of different cell types per genome. The abundance is the number of proteins in a genome that contain at least one domain of a particular superfamily. The abundance profile is the collection of abundances of a domain superfamily across several genomes. In normalised form, the profile expresses relative domain abundances. 

The Pearson correlation coefficient *R* is a measure of linear relationship between to sets of variables; *R* equals 1 or −1 if there is a perfect positive or negative linear correlation, respectively. |*R*| ≤ 0.20 implies a very weak or nonexistent linear correlation. Squared *R (R^2^)* is a measure for the proportion of variance in the data that are explained by a linear relationship between two variables, e.g. about two thirds of the variance are explained at *R* = 0.80 (*R^2^* = 0.64).

**Figure 2:**
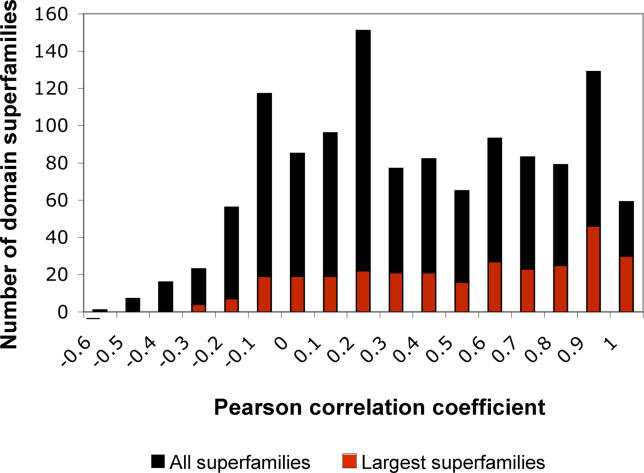
Some Family Expansions Correlate Well with the Number of Different Cell Types in Each Organism For each of the 1,219 domain superfamilies and their profile of abundance in the 38 genomes, we calculated the correlation coefficient *R* of the profile with the number of different cell types per organism. The distribution of *R* values is plotted in black. For the subset of largest superfamilies (i.e., those with at least 25 proteins in one of the genomes) the distribution of *R* values is shown in red. There are few superfamilies with high correlation (*R* ≥ 0.80), and many with poor correlation or slight anticorrelation (*R* ≤ 0.20); this distribution is similar for both sets of superfamilies.

The distribution of correlation coefficients *R* between abundance profiles and the number of different cells types is shown in Figure 2. Only 15% of the superfamilies (194/1,219) show a strong correlation between their abundance and the number of different cell types per organism, with *R* ≥ 0.80. These superfamilies expand mostly in vertebrates, have intermediate abundance in other animals and plants, and are of low frequency or absent in fungi and protozoa. Some 40% of the superfamilies (470/1,219) have a correlation coefficient *R* between 0.20 and 0.80. Close to 45% of the superfamilies (555/1,219) have correlation coefficients between 0.20 and negative values.

### Family Expansions with Good Correlation with the Number of Cell Types

**Figure 3:**
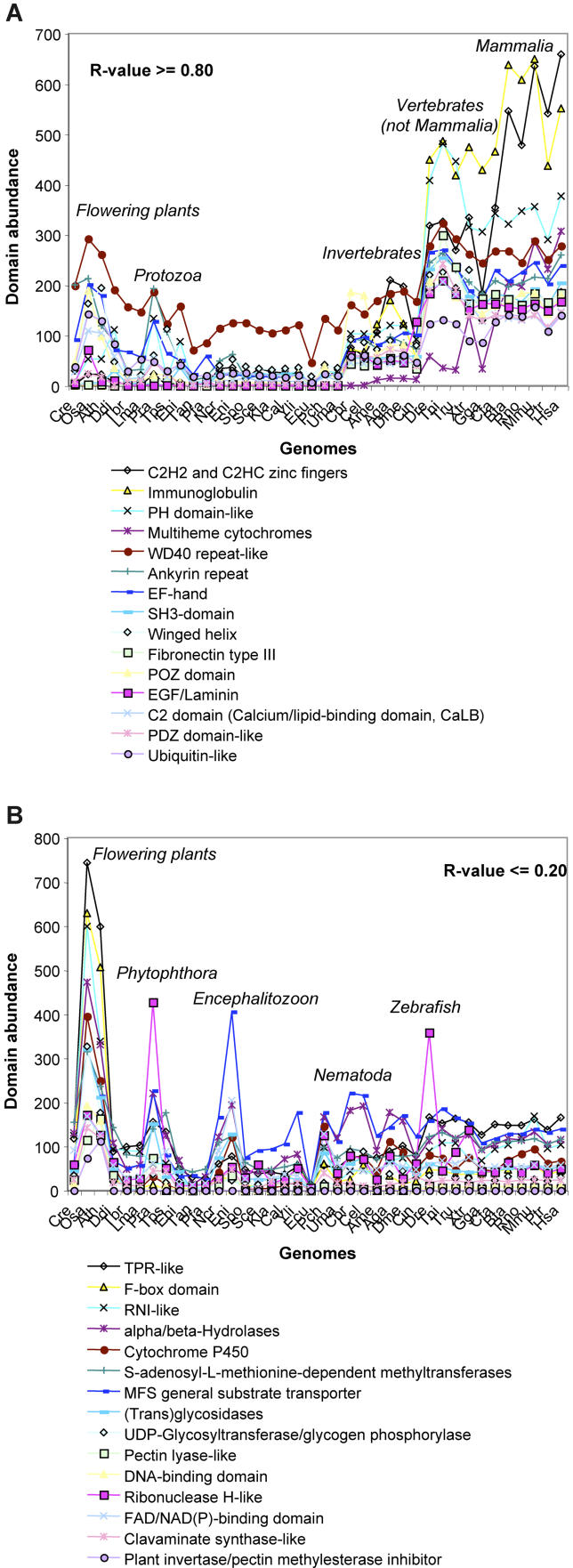
Examples of Family Expansions with Good or Poor Correlation with the Number of Different Cell Types There are 194 superfamilies with good (*R* ≥ 0.80; [A]) and 555 superfamilies with poor or negative (*R* ≤ 0.20; [B]) correlation with the number of different cell types, and the diagrams shows 15 examples of each. Some of the peaks are annotated in italics. The genomes are in the same order as in Figure 1A. The lines between counts of domain abundance are for better visualisation only. Abbreviations are as in Figure 1.

We examined in detail the properties of those superfamilies that have strong correlations with the number of different cell types in 38 organisms (*R* ≥ 0.80). These proteins are described in Tables 1–3, and Figure 3A. This group of superfamilies represents only 15% of all superfamilies (194/1,219), but they are found in more than 40% of human domains (7,825/19,225; Table 3). In contrast, they form only a tenth of the domains in *Arabidopsis* (1,884/19,323; Table 3).

**Table 2:**
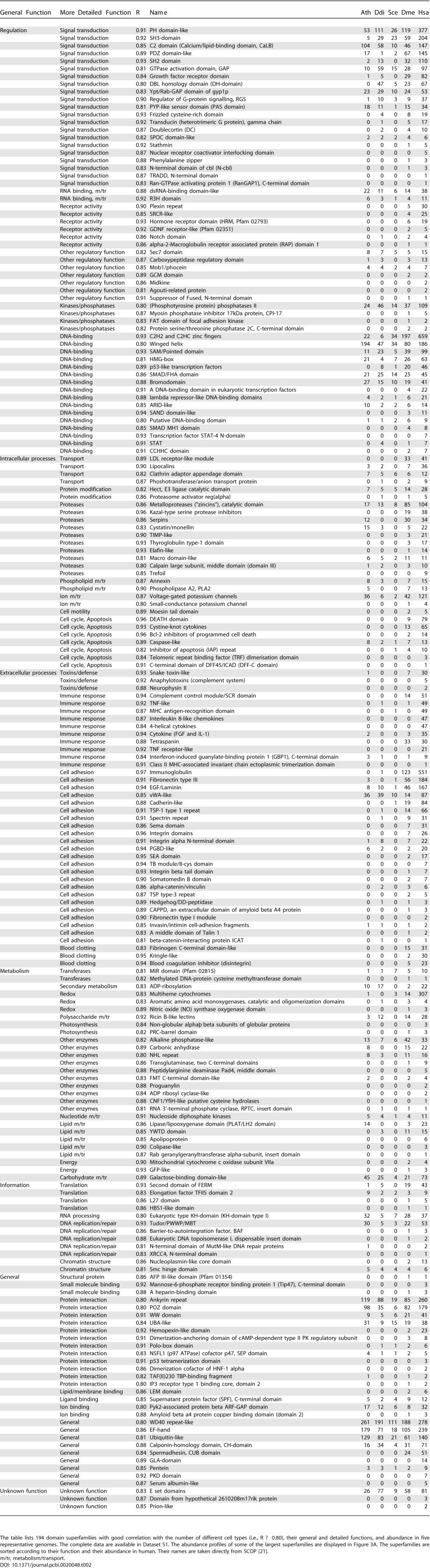
Domain Families with Good Correlation with the Number of Different Cell Types

Generally, we can distinguish three types of expansion patterns among the 194 superfamilies with high correlations (Table 2 and Dataset S1). Close to one-third of the superfamilies are found in all 38 genomes, one-third exclusively occurs in animals, and one-third occurs in animals and has a spasmodic distribution in the other kingdoms. Of those found in all genomes, the abundance is usually highest in vertebrates, particularly in mammals, and moderate in the other animals and low in plants, protozoa, and fungi. Examples are the PH domains and GTPase activation domains, which both function in signal transduction (Table 2). Another example is voltage-gated potassium channels, whose vertebrate-specific expansion is possibly linked to their function in neural signalling. Two-thirds of the 194 superfamilies are only commonly found in animals, but are absent or occur in very low frequencies in the other kingdoms (Table 2). Examples are tumour necrosis factor (TNF)–like, TNF receptor–like, and also DEATH domains, all of which are known to function in apoptosis. Some 12% of these superfamilies are vertebrate specific, and examples of these include proteins of the immune system, such as major histocompatibility complex antigen–recognition domains, or four-helical cytokines.

### Family Expansions with No or Inverse Correlation with the Number of Cell Types

There are 555 of the 1,219 superfamilies whose abundances have correlation coefficients of less than 0.20; examples of these families are shown in Figure 3B. Most of the expansions do not correlate with the number of different cell types (|*R*| ≤ 0.20). Only 95 superfamilies show weak inverse correlation (*R* ≤ −0.20), and these superfamilies are usually small (see Dataset S1). No superfamily expansion displays strong negative correlation (*R* ≤ − 0.80). For this reason we regard all superfamilies with *R* ≤ 0.20 as one group.

Domains from these superfamilies are found in 39% of the domains in *Arabidopsis* (7,620/19,323), but only 11% of the domains in humans (2,206/19,225; Table 3). Similar to the well-correlated superfamily expansions described above, the superfamily expansions with little correlation to the number of different cell types are significantly biased in their functions (*p* < 0.001). The major contributions to this set of superfamilies come from metabolism (45%) and information storage and replication (16%); smaller contributions are made by the other function categories (Table 1).

In many cases, the distribution of these superfamilies follows one of three patterns. They (1) generally occur in very small numbers with a spasmodic distribution (167 superfamilies seen in a total of ten or fewer proteins); (2) are widely spread with low frequencies (about half of the superfamilies occur in ≤5 proteins/organism on average); or (3) have expansions in one or a few genomes and low frequencies elsewhere. Many of these expansions have their highest abundance in plants, and intermediate or low numbers in animals.

Some examples of these superfamilies are shown in Figure 3B, sorted with respect to their abundance in *Arabidopsis.* Large superfamilies include Tetratricopeptide-repeat–like or F-box protein interaction domains that are typical for *Arabidopsis* and often involved in ubiquitination [30], and many enzymes. Another large superfamily in *Arabidopsis* is the ribonuclease inhibitor–like domains that are leucine rich and known to inhibit ribonucleases but also to bind other proteins and function in nucleic acid processing [30]. Thus they may also have a role in RNA interference, a process that is common in plants [31].

### Correlated Expansions Show Three Major Trends

**Figure 4:**
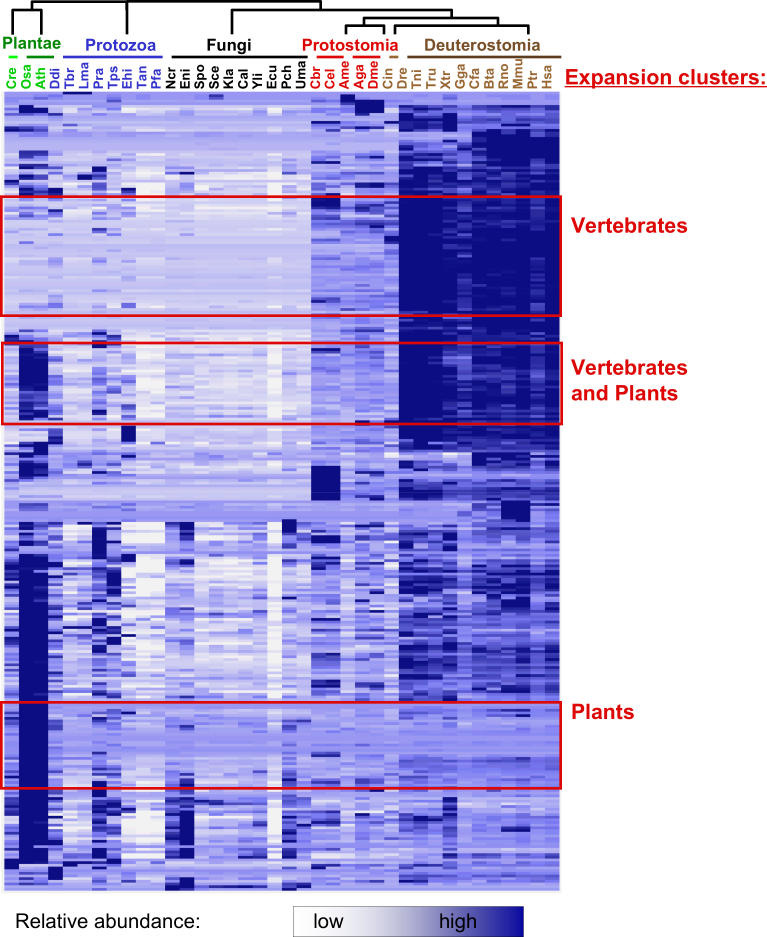
Domain Superfamilies Show Different Expansion Patterns The matrix shows the 299 largest domain superfamilies that occur in ≥25 proteins in at least one of the genomes, hierarchically clustered. Each row represents one superfamily. Colour-coded profiles show the normalised abundance of each domain superfamily across the different eukaryotic genomes: white, low relative abundance; blue, high relative abundance. Each column represents one genome. All genomes are abbreviated and organised as in Figure 1A. A grouping of superfamily pairs with *R* ≥ 0.90 results in 26 clusters, and the three largest clusters are indicated in red boxes: expansions in vertebrates (52 superfamilies) and expansions in plants (33 superfamilies), and expansions in vertebrates and plants (26 superfamilies). Further descriptions can be found in Table 4 and at http://polaris.icmb.utexas.edu/people/cvogel/HV.

One implication of our work is an evaluation of the correlation between domain superfamily expansion profiles (i.e., an identification and description of the different duplication patterns of duplications that formed eukaryotic protein repertoires). To do so, we calculated the correlation coefficient (*R* value) for each pair of relative abundance profiles for the 299 largest superfamilies and then grouped the families sharing a high *R* value. The result of such clustering is shown in Figure 4. In the figure, each row denotes a domain superfamily; each column denotes a particular genome. The relative abundance of the domain superfamily in each genome is colour-coded, and the abundance profiles are hierarchically clustered.

**Table 3:**
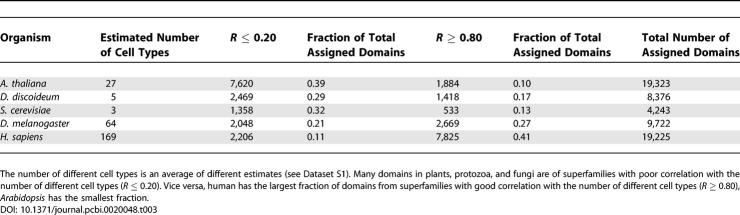
Contribution of Different Groups of Domain Superfamilies to the Overall Composition of Genomes

**Table 4:**
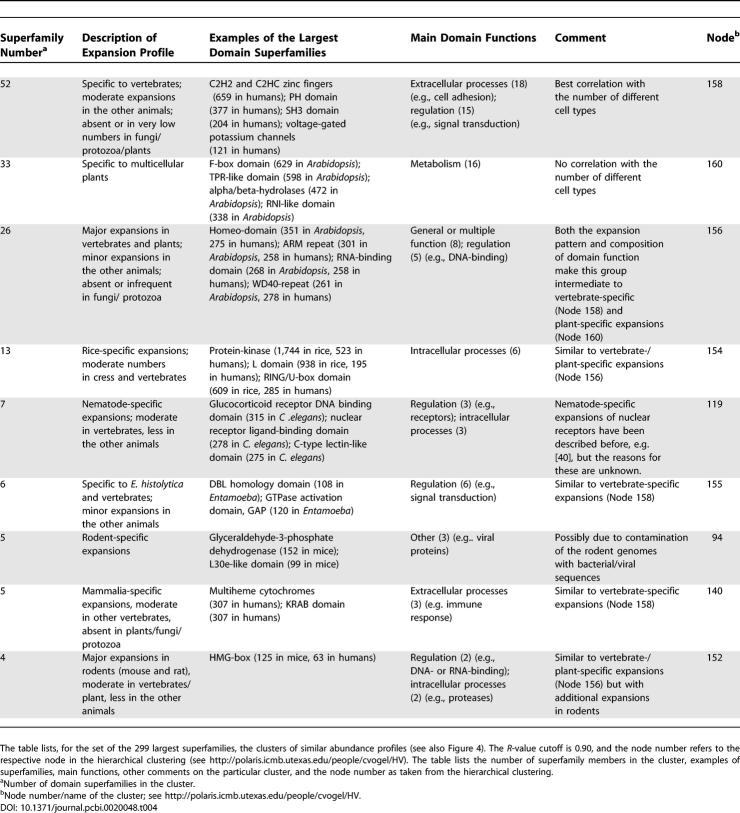
Patterns of Domain Superfamily Expansions

We examined the functions of the domain superfamilies to understand how their duplications may have supported the emergence of novel cell types. For the 194 superfamilies with good correlation with the number of different cell types, all functional categories make some contribution, but two make disproportionally large ones (*p* < 0.001; Tables 1 and 2). These categories include superfamilies of extracellular processes (20%) and superfamilies of regulation (29%), encompassing close to one-half (49%) of the 194 superfamilies. In particular, these families include many domains of signal transduction (e.g., PH-, SH3, and SH2 domains), DNA-binding domains (e.g., C2H2 and C2HC zinc fingers, and winged helix domains), kinases and phosphatases and cell adhesion molecules (e.g., immunoglobulin, fibronectin type III, or EGF/laminin domains) (Table 2). We also observe several large families (e.g., dsRNA-binding, Tudor/PWWP/MBT, SAM/Pointed, or KH domains) that are known to have RNA-binding activity [30], some in addition to DNA-binding activity. The other function contributions usually include smaller superfamilies, and consist of domains involved in metabolism, intracellular processes, and information (Tables 1 and 2).

When applying an *R*-value cutoff of 0.90, we obtained 26 clusters with correlated abundance profiles, and all clusters with four or more members are described in Table 4. We observed three major trends of domain superfamily expansions and several trends with fewer superfamilies involved. One-sixth of the domain superfamilies (52/299) expand specifically in vertebrates, have intermediate abundance in the other animals and plants, and very low abundance in protozoa and fungi. Unsurprisingly, all of these superfamily expansions, except for one, belong to the 194 expansions described above as strongly correlated with the number of different cell types. Further, we observed a group of 26 superfamilies that expand in both plants and vertebrates as compared to other organisms, and have intermediate numbers in the other animals. A third group of 33 superfamilies expand in plants, but have very low abundance in all other organisms.

In contrast to these three major trends, most superfamilies do not belong to the groups of vertebrate- and/or plant-specific expansions, but are members of 23 smaller clusters that have expansions specific to one or few genomes other than plants or vertebrates. These families are often of low abundance. The contribution of these superfamilies to the protein repertoire confirms previous findings on the prevalence of “lineage-specific gene family expansions” that is the emergence of different domain architectures, through domain accretion and domain shuffling, in different phylogenetic lineages [10,11,32–34].

We conducted similar clustering with all 1,219 superfamilies and a range of *R*-value cutoffs (see Protocol S1 and Figure S2). The results for these procedures are qualitatively the same as those described above. A more detailed description of the different expansion patterns (Figure 4 and Table 4) is very interesting, but goes beyond the scope of this paper and will be left to future analysis.

### Future Refinements of Our Work

In the work described here we have often given precise numbers and descriptions. For a variety of reasons we expect that these statements will be refined or modified in future research. First, gene predictions, especially of recently sequenced genomes, often contain some errors. For example, we noticed a large number of *Xenopus-*specific expansions (59 superfamilies, *R* ≥ 0.80), many proteins of which may in fact come from erroneously included bacterial sequences. Second, the prediction of protein domains is obviously biased by our current knowledge of domain structure, and only the completion of the systematic structural genomics projects will provide an accurate survey of domain distributions across genomes. Currently we are able to predict these domains in ~60% of the sequences [18]; increases in coverage will improve our knowledge, especially about smaller protein families. Third, we have used here rough general annotations of the functions carried out by members of different superfamilies. This annotation needs to be refined, and new experimental characterisation will reveal further functional variety within domain superfamilies. Last but not least, while the number of different cell types is a valid measure of organismal complexity, it crucially depends on how these cell types are defined [28,29]. These definitions still need to be improved, although first, most valuable steps have already been taken [35]. However, given these qualifications, we would expect that future work will not upset the broad conclusions derived by our analysis.

### Summary and Conclusions

We present here one of the first studies that directly links protein family expansions to increases in eukaryotic complexity. We go beyond what has been known before in several ways. First, we use the number of different cell types as a measure of biological complexity. Second, we address a larger number of eukaryote genomes than previous analyses: we particularly focus on 17 completely sequenced animals, including two nematodes, three insects, one urochordate, and 11 vertebrates, and compare them to the genomes of three plants, eight protozoa, and ten fungal genomes (Figure 1). Third, as the proteins of these distantly related organisms are highly diverged, we include information on protein structure to accurately determine the family relationships [16,36], using domains as the smallest structural, functional, and evolutionary unit.

We demonstrate that, independent of the total number of genes, particular protein families expand in concert with increases in biological complexity and functions of these superfamilies can be linked to the evolution of more intricate physiological features. These family expansions are largely caused by gene duplications rather than by domain accretion [33] or by invention of new superfamilies: the average protein lengths, which indicate the average number of domains per protein, are similar for all 38 eukaryotes (Figure S3). As domain reshuffling is known to be linked to domain duplication [37,38], domain superfamily expansions also provide the basis for an increase in the number of domain combinations and multidomain proteins in vertebrates [13,39], which in turn increases proteome complexity.

Our work suggests that the two basic types of duplication have different relative contributions to proteomes. “Conservative expansions” do not correlate with an increase in the number of different cell types, but simply enlarge the genome size. Most protein families belong to this group. The functions of the domains involved define organism-specific properties [10,11,32,34]: they help the organism to adapt to environmental challenges. Examples are the expansions of chemoreceptors in nematodes [40] and metabolic domains in plants whose expansions correlate with an increase in the number of secondary metabolites.

In contrast, “progressive expansions” correlate with an increase in physiological complexity, but they represent only a small fraction of all domain superfamilies. We identify ~200 domain superfamilies that are both correlated with each other in their abundance pattern and correlate with the number of different cell types in eukaryote organisms; these are candidates for progressive expansions. Domains of these expansions are likely to have enabled the emergence of novel cell types and the communication between these cells [41], for example, by their functions in extracellular, or regulatory processes. An increased number of cell adhesion molecules, but also apoptotic domains, supports the intricate embryonic development found in animals. Large families of transcription factors or proteins with protein-binding domains result in complex intra- and intercellular signalling and regulatory networks. Further, the expansion of some protein families correlates with the emergence of animal- or vertebrate-specific traits, such as the immune system. Finally, some families (e.g., RNA-binding domains) may support regulation of alternative splicing that plays a significant role in humans [42], and, together with other post-transcriptional and -translational modifications, further increase the complexity of vertebrate proteomes.

In general, plants and animals went separate paths with large, kingdom-specific expansions. We observed, however, some plant-specific expansions, which also occurred in vertebrates, but were not as pronounced in the other animals. These patterns may correlate with macroscopic features yet to be identified. For example, future work may include measures such as the number of cells, the body mass, or even population size [43] in a description of organismal complexity.

Finally, we hope that our analysis provides a framework for more detailed studies of family expansions. One example for such studies is domains of the immunoglobulin superfamily that expanded in number in *Drosophila* as compared to *C. elegans.* Most of the fly-specific proteins have been shown to be cell-surface receptors and cell-adhesion molecules that are involved in axon pathfinding during the embryonal development of the nervous system [15,44,45]—this illustrates one of the factors that allows the fly to have a more complex nervous system than the worm.

## Materials and Methods

### Datasets.

The 38 eukaryotic genomes used in our analysis are listed in Figure 1A, and our analysis is outlined in Figure 1B. The gene predictions and domain assignments to the gene predictions were taken from the SUPERFAMILY database version 1.69, updated in September 2005 [18]; information on genome versions and source can be found at http://supfam.mrc-lmb.cam.ac.uk/SUPERFAMILY. The domain superfamilies are defined in the SCOP database [21], and our analysis focuses on the seven well-defined classes *a* to *g,* respectively. All domains within a SCOP superfamily are related and can be regarded as descendants from one common ancestral domain. The *Arabidopsis* and animal genomes were made nonredundant with respect to predicted splice variants: for each gene only the longest transcript was included. Information on predicted splice variants was unavailable for the fungal genomes (except for S. cerevisiae), *Oryza sativa,* and the protozoa. Information on the estimated number of different cell types was taken from literature [28,29] and is detailed in Dataset S1.

### Correlations between superfamilies and the number of different cell types.

The abundance of a domain superfamily in each genome was measured as the number of proteins with at least one predicted hit of the respective superfamily (Figure 1 and Dataset S1). Many domain superfamilies occur in only one or two genomes and in only a few proteins. For each superfamily, changes in abundance across different eukaryotes can be described in an abundance pattern or profile. The abundance counts for one superfamily across different genomes were normalised according to *A_n = (A_i – A_avg) / A_sdv,* where *A_i* and *A_n* are the absolute and normalised abundance count in a particular genome, respectively, and *A_avg* and *A_sdv* are the average abundance and standard deviation across all genomes for that superfamily, respectively. This means the abundance of a superfamily in one genome is described relative to its abundance in other genomes.

Similar to what has been done for gene expression data in other studies, each expansion pattern was colour-coded, using the matrix2png [46] and treeview programs (http://rana.lbl.gov/EisenSoftware.htm) for visualisation. In Figure 4, each row depicts the profile for one superfamily: blue denotes high, and white denotes low relative abundance. The genomes are arranged in the same order as in Figure 1A, and the rows (superfamilies) are hierarchically clustered using the XCluster software (http://genetics.stanford.edu/~sherlock/cluster.html). A cutoff of *R* ≥ 0.90 results in clusters of highly similar expansion patterns with largest clusters indicated in red (Figure 4). The clusters are also described in Table 4 and at http://polaris.icmb.utexas.edu/people/cvogel/HV.

Similar to correlating the abundance profiles of superfamilies with each other, we also correlated them with the number of different cell types per organism. In an extension of what we published previously [38], we assigned each domain superfamily to one of 50 small functional categories (see Protocol S1). Each of the small categories maps to one of seven larger functional categories.

## Supporting Information

Dataset S1 Characteristics of the 38 Genomes and the 1,219 Superfamilies The spreadsheet genome_characteristics lists the 38 genomes used in our analysis, as taken from SUPERFAMILY version 1.69 [18], http://supfam.mrc-lmb.cam.ac.uk/SUPERFAMILY. Please refer to the SUPERFAMILY database for further information on the origin of the genome sequences. The two-letter abbreviation for each genome is the one used in the SUPERFAMLY MySQL database. The three-letter abbreviation for each genome is used in our paper. The information on the total number of genes (non-redundant in terms of splice variants), average gene length, and the total number of domains predicted by SUPERFAMILY (domains of all classes) were taken directly from the SUPERFAMILY database [18].
The estimated number of different cell types are taken from the publication by Valentine et al. [28] and Hedges et al. [29]. The average of these values represents the estimated number of different cell types used in this analysis.
The spreadsheet superfamily_data contains information on the abundance of the 1,219 superfamilies in 38 genomes. The superfamilies are annotated in terms of their general and more detailed type of function, their identifier used in the SCOP [21] and in the SUPERFAMILY [18] database, and their correlation with the estimated number of different cell types.
(982 KB XLS)

Figure S1 Distributions of Domain Functions (A) Distribution of functions in terms of domain superfamilies defined in SCOP [21]. Domain superfamilies of metabolism (e.g., enzymes) are the most abundant category. (B) shows the distribution of superfamilies across the function categories; this distribution is similar for all genomes, five of which are shown. This means that invention of domain superfamilies specific to some genomes did not significantly change the overall composition in terms of function. This is different when taking gene duplication into account (C): the composition in terms of domain functions varies within the five genomes shown. While the largest category in plant is metabolism, in human it is regulation.
Previous work reported a linear relationship between genome size and the number of metabolic proteins for bacteria and eukaryotes [8,9]. Such a linear relationship would result in a constant fraction of metabolic domains across genomes, but this is not what we observe when comparing five different eukaryotes (D): the fraction of domains in metabolism is lower in invertebrates and vertebrates (fly and human) than in the other organisms. These differences observed may be due to different datasets (domains used instead of whole proteins) and different function annotation procedures.
Abbreviations are as in Figure 1.
(46 KB PDF)

Figure S2 Expansion Profiles of all 1,219 Superfamilies Similar to Figure 4, the matrix displays the relative abundance profiles for each of the 1219 superfamilies (rows) in the 38 genomes (columns) in a colour-coded format. Blue denotes high, and white denotes low relative domain abundance in some organisms as compared to others. As for the subset of 299 largest superfamilies (Figure 4), three major trends become apparent: expansions specific to vertebrates, expansions specific to plants, and expansions that occur in plants and vertebrates.
Abbreviations are as in Figure 1.
(678 KB TIF)

Figure S3 Relationship between the Number of Different Cell Types, Total Number of Domain Superfamilies, Total Number of Domains per Genome, and Sequence Length The number of different cell types is only weakly correlated with the number of different domain superfamilies found (*R^2^* = 0.52, [A]), the total number of genes predicted for an organism (*R^2^* = 0.54, Figure 1A), and with the total number of domains (*R^2^* = 0.59, [B]). Part of the latter correlation can be explained by the fact that more domains are known and assigned to vertebrates than to protists and plants. There are no large differences in the average sequence length of fungi, protists, plants, or vertebrates (*R^2^* = 0.02, [C]). Thus, the higher number of domains in some organisms as compared to others must largely arise from duplication of whole genes rather than the addition of domains to existing proteins.
The number of different domain superfamilies can be taken as a measure of invention of novel families in an organism, while the total number of domains is a measure of duplication. Thus, duplication correlates better than invention with increases in biological complexity as measured in the number of different cell types, and may have been one of the driving forces behind the emergence of novel cell types.
Abbreviations are as in Figure 1.
(64 KB PDF)

Protocol S1 Notes on Domain Function Annotation and Clustering Procedure (144 KB PDF)

Table S1 Summary of Key Terms Used in the Paper (109 KB DOC)

Table S2 Groups of Domain Function and the Number of Different Cell Types Domains in the function categories are non-overlapping subsets of all domains in each organism. Only two function categories (i.e., domains of extracellular processes and regulation) show very good correlation of domain abundance and the number of different cell types (i.e. *R* ≥ 0.80).
(110 KB DOC)
Additional supporting material can be found at http://polaris.icmb.utexas.edu/people/cvogel/HV. The Web site contains several files: (1) mapping of the 50 more detailed function categories to the seven main function categories; and (2) names, SCOP identifiers, and SUPERFAMILY identifiers of all SCOP superfamilies, v. 1.69 [18]. The Web site also has links to additional Web pages, which display clusterings of superfamily expansion profiles using different cutoffs. Each of the Web pages describes clusters of similar expansion profiles, and the number and size of the clusters depends on the cutoffs used and the distribution of domain functions. Each cluster is labelled with a unique node number, and this number is taken directly from output of the XCluster program at http://genetics.stanford.edu/~sherlock/cluster.html.

## Abbreviations

SCOP: Structural Classification of Proteins
TNF: tumour necrosis factor
