# Supplementary figures and images for: Protein Family Expansions and Biological Complexity
Source: PLoS Comput Biol. 2006 May 26;2(5):e48. doi: 10.1371/journal.pcbi.0020048 (PMC1464810; doi:10.1371/journal.pcbi.0020048)

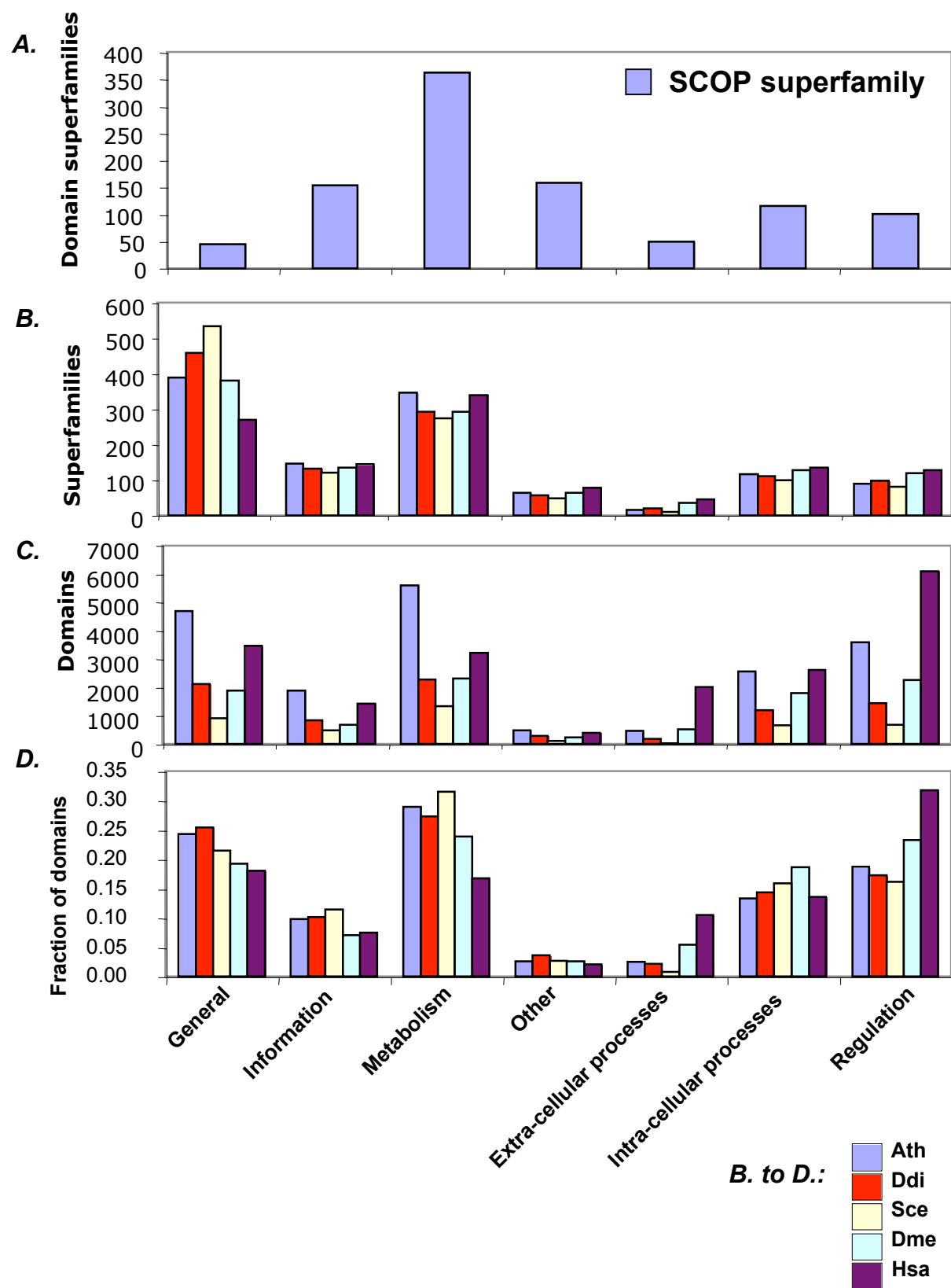

Supplement: Figure S1 — (A) Distribution of functions in terms of domain superfamilies defined in SCOP [21]. Domain superfamilies of metabolism (e.g., enzymes) are the most abundant category. (B) shows the distribution of superfamilies across the function categories; this distribution is similar for all genomes, five of which are shown. This means that invention of domain superfamilies specific to some genomes did not significantly change the overall composition in terms of function. This is different when taking gene duplication into account (C): the composition in terms of domain functions varies within the five genomes shown. While the largest category in plant is metabolism, in human it is regulation. Previous work reported a linear relationship between genome size and the number of metabolic proteins for bacteria and eukaryotes [8,9]. Such a linear relationship would result in a constant fraction of metabolic domains across genomes, but this is not what we observe when comparing five different eukaryotes (D): the fraction of domains in metabolism is lower in invertebrates and vertebrates (fly and human) than in the other organisms. These differences observed may be due to different datasets (domains used instead of whole proteins) and different function annotation procedures. Abbreviations are as in Figure 1. (46 KB PDF) [file pcbi.0020048.sg001.pdf]

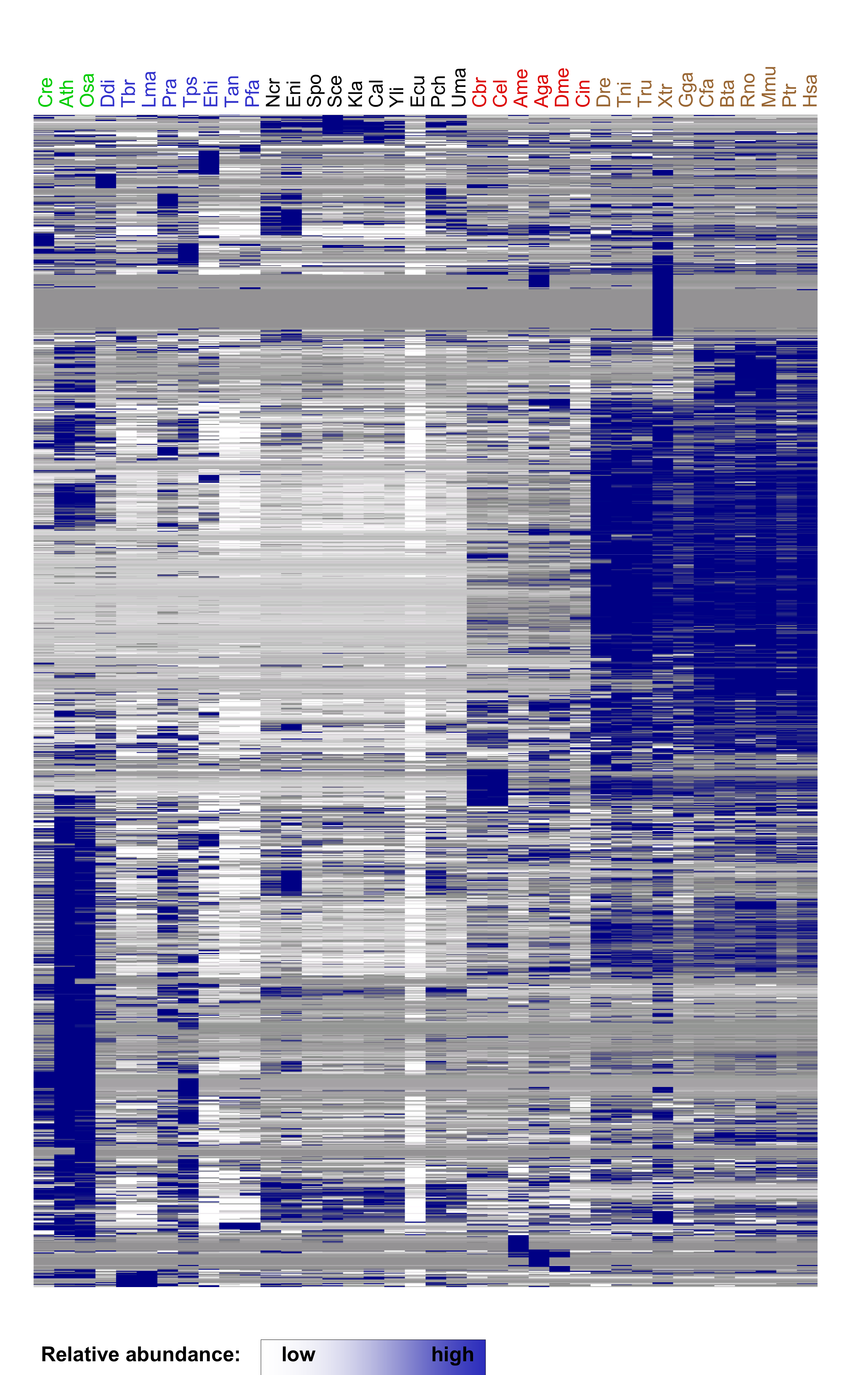

Supplement: Figure S2 — Similar to Figure 4, the matrix displays the relative abundance profiles for each of the 1219 superfamilies (rows) in the 38 genomes (columns) in a colour-coded format. Blue denotes high, and white denotes low relative domain abundance in some organisms as compared to others. As for the subset of 299 largest superfamilies (Figure 4), three major trends become apparent: expansions specific to vertebrates, expansions specific to plants, and expansions that occur in plants and vertebrates. Abbreviations are as in Figure 1. (678 KB TIF) [file pcbi.0020048.sg002.tif]

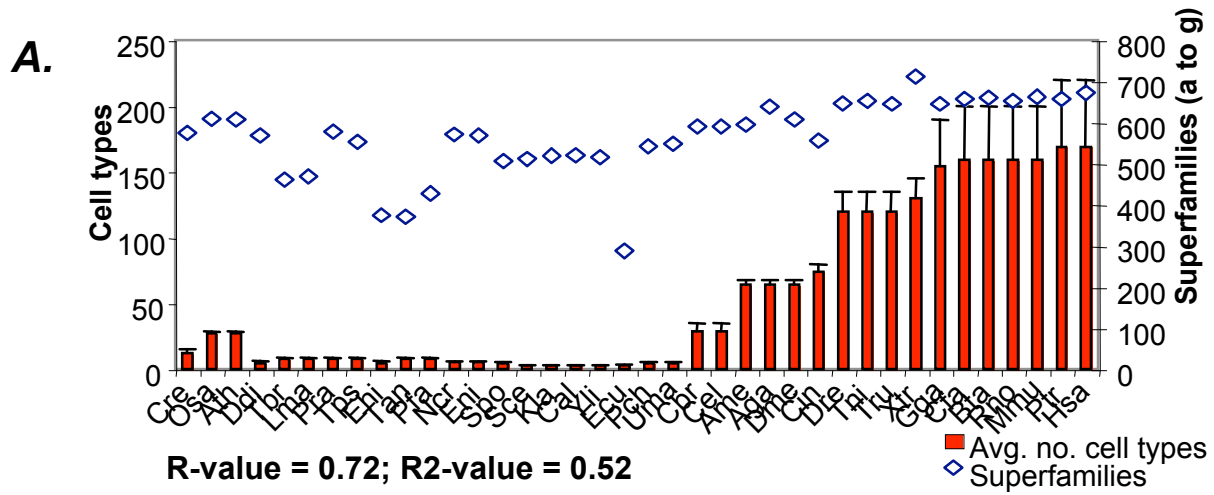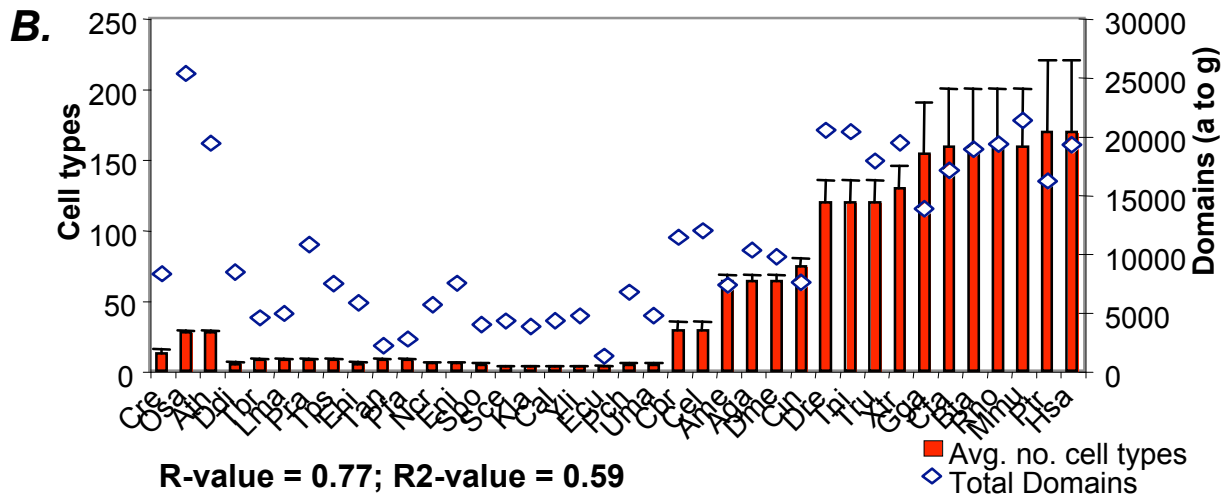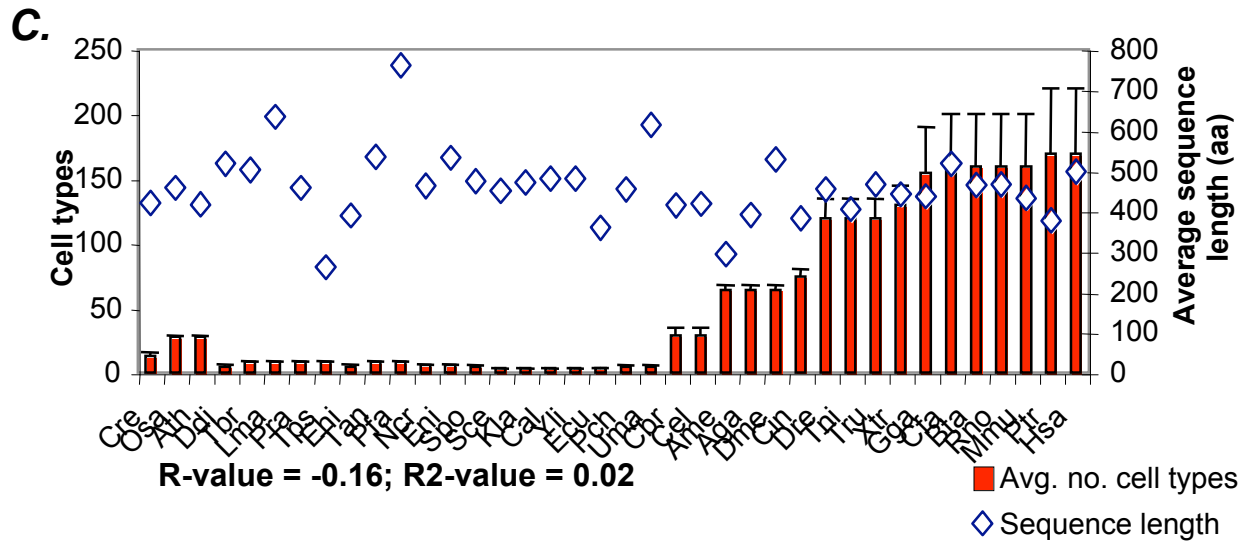

Supplement: Figure S3 — The number of different cell types is only weakly correlated with the number of different domain superfamilies found (R2 = 0.52, [A]), the total number of genes predicted for an organism (R2 = 0.54, Figure 1A), and with the total number of domains (R2 = 0.59, [B]). Part of the latter correlation can be explained by the fact that more domains are known and assigned to vertebrates than to protists and plants. There are no large differences in the average sequence length of fungi, protists, plants, or vertebrates (R2 = 0.02, [C]). Thus, the higher number of domains in some organisms as compared to others must largely arise from duplication of whole genes rather than the addition of domains to existing proteins. The number of different domain superfamilies can be taken as a measure of invention of novel families in an organism, while the total number of domains is a measure of duplication. Thus, duplication correlates better than invention with increases in biological complexity as measured in the number of different cell types, and may have been one of the driving forces behind the emergence of novel cell types. Abbreviations are as in Figure 1. (64 KB PDF) [file pcbi.0020048.sg003.pdf]
