# Supplementary material for: Protein Family Expansions and Biological Complexity
Source: PLoS Comput Biol. 2006 May 26;2(5):e48. doi: 10.1371/journal.pcbi.0020048 (PMC1464810; doi:10.1371/journal.pcbi.0020048)
Supplement: Protocol S1 — (144 KB PDF) [file pcbi.0020048.sd002.doc]

# Supporting information --

# Protein family expansions and biological complexity

Christine Vogel^1,2^,* and Cyrus Chothia^1^

^1^MRC Laboratory of Molecular Biology, Hills Road, Cambridge CB2 2QH, England

^2^Institute for Cellular and Molecular Biology, University of Texas at Austin, 2500 Speedway, MBB 3.210, Austin, TX 78712, USA

*Corresponding author: cvogel@mail.utexas.edu

**Abbreviations:** HMM – hidden Markov model; R-value – Pearson correlation coefficient; R^2^ – squared R-value

# Supporting Protocol S1

## Domain function annotation

The domain superfamilies were annotated with respect to their function as described previously [1,2]. The annotation was updated for the latest release of the SCOP database, and extended to all classes *a* to *g*. The annotation was based on information in SCOP [3], Interpro [4,5] and literature. The function annotation is available in the **Supporting Dataset S1**, please cite us if you use it [1,2].

As a control, we used the automated annotation of GO process, function and location to Pfam domains available in InterPro [4]. Pfam domains were mapped onto SCOP domain superfamilies based on sequence similarity. This provided annotation for 647, 667 and 343 domain superfamilies, respectively. The manual domain annotation was largely consistent with the Gene-Ontology annotation [6] for Pfam domains [4,7] and their mappings to the domains described for SUPERFAMILY [8].

## Clustering expansion profiles: optimising the clustering procedure

In order to be able to compare the expansion profiles of domain superfamilies the abundance profiles were normalised as described in the main text. While the exact numbers change depending on different parameters used (see below), the overall results are the same independent of the cutoffs: about one-third to half of the domain superfamilies belong to three major expansion profiles.

We tested the following modifications:

a) Measuring abundance in alternative ways, e.g. as the total number of domains, not proteins, returned similar results. This is because most proteins contain only one instance of a particular domain superfamily.

b) Our analysis includes the 299 domain superfamilies which have at least 25 occurrences in at least one of the genomes (**Figure 4**, main text). Lower and higher abundance cutoffs, e.g. 20 or 30 minimum abundance, resulted in three main clusters similar to those described for the cutoff of 25 proteins. When including all superfamilies (minimum abundance = 1), we also obtained similar results (**Supporting website** and **Figure S3**): three major trends govern the expansion of protein families in eukaryotes.

At low abundance cutoffs (<=20), more superfamilies were included, but the abundance of these superfamilies contributes only little to the proteome, and the abundance profile describing the extent of duplication is little meaningful. At high abundance cutoffs (>=40), the abundance profiles have a stronger functional bias, but also fewer superfamily members and are difficult to evaluate statistically. Thus, the cutoff used in our paper, i.e. abundance minimum of 25 proteins, is the result of balancing between including as many superfamilies as possible, and still obtaining useful cluster sizes.

c) We also tested other clustering algorithms (self-organising maps), a different distance measure (Euclidean distance) and other normalisation methods.

The results were always similar (data not shown). For all different sets of parameters, we always needed to compromise between i) a sufficiently large number of superfamilies in our dataset; ii) obtaining meaningful abundance profiles; iii) the need to have sufficiently large clusters so that the signal within a cluster was clear enough, and to still obtain well-separated clusters. The results of different thresholds for the Pearson Correlation Coefficient (R-value) are shown on the **Supporting website**.

# References

1. Vogel C, Berzuini C, Bashton M, Gough J, Teichmann SA (2004) Supra-domains - evolutionary units larger than single protein domains. J Mol Biol 336: 809-823.

2. Vogel C, Teichmann SA, Pereira-Leal JB (2005) The relationship between domain duplication and recombination. J Mol Biol 346: 355-365.

3. Andreeva A, Howorth D, Brenner SE, Hubbard TJ, Chothia C, et al. (2004) SCOP database in 2004: refinements integrate structure and sequence family data. Nucleic Acids Res 32: D226-229.

4. Mulder NJ, Apweiler R, Attwood TK, Bairoch A, Barrell D, et al. (2003) The InterPro Database, 2003 brings increased coverage and new features. Nucleic Acids Res 31: 315-318.

5. Mulder NJ, Apweiler R, Attwood TK, Bairoch A, Bateman A, et al. (2005) InterPro, progress and status in 2005. Nucleic Acids Res 33: D201-205.

6. Harris MA, Clark J, Ireland A, Lomax J, Ashburner M, et al. (2004) The Gene Ontology (GO) database and informatics resource. Nucleic Acids Res 32: D258-261.

7. Bateman A, Coin L, Durbin R, Finn RD, Hollich V, et al. (2004) The Pfam protein families database. Nucleic Acids Res 32: D138-141.

8. Madera M, Vogel C, Kummerfeld SK, Chothia C, Gough J (2004) The SUPERFAMILY database in 2004: additions and improvements. Nucleic Acids Res 32: D235-239.
