# Supplementary material for: Protein Family Expansions and Biological Complexity
Source: PLoS Comput Biol. 2006 May 26;2(5):e48. doi: 10.1371/journal.pcbi.0020048 (PMC1464810; doi:10.1371/journal.pcbi.0020048)
Supplement: Table S1 — (109 KB DOC) [file pcbi.0020048.st001.doc]

## Table S1. Key terms

The box lists the most important terms that are used in our analysis.

| **Term** | **Definition / Description** |
| --- | --- |
| **protein** | Amino acid product from genes; consists of one or several domains; in this work, only one protein per gene is counted, disregarding additional splice variants |
| **domain** | Structural, functional and evolutionary unit of proteins; defined in the SCOP database [1] |
| **protein family** | Grouping of proteins which contain at least one domain of common descent, i.e. the same superfamily |
| **superfamily** | Grouping of distantly related proteins whose structure, function and sequence features imply homologoy; defined in the SCOP database [1]; two proteins with a domain of the same superfamily are grouped into a protein family |
| **largest superfamilies** | Subset of domain superfamilies that are highly abundant, i.e. that occur in at least 25 proteins in at least one of the genomes in our analysis |
| **abundance** | Number of proteins in one genome which contain a domain of a particular domain superfamily, i.e. the number of paralogous domains; denotes the size of a domain superfamily or protein family |
| **(abundance) profile** | Arrangement/pattern of abundances of a domain superfamily in several genomes; also used in normalised form to express the relative abundance of the domain superfamily in the genomes |
| **R** | Pearson correlation coefficient; measure of the linear correlation between to sets of variables; the R-value equals 1 or -1 if there is a perfect positive or negative linear correlation, respectively; an R-value<=|+-0.20| implies a very weak or non-existent linear correlation; note: some of our analyses describe superfamilies with R-value<=0.20 which includes both those that are not correlated and those that are inversely correlated with the number of different cell types |
| **R^2^** | Squared R-value; measure for the proportion of variance in the data that are explained by a linear relationship between the two variables; for example, an R-value of 0.80 implies that almost two thirds (R^2^=0.64) of the data variance correlate is explained by a linear model |

**Reference**

1. Andreeva A, Howorth D, Brenner SE, Hubbard TJ, Chothia C, et al. (2004) SCOP database in 2004: refinements integrate structure and sequence family data. Nucleic Acids Res 32: D226-229.
