# Supplementary material for: Protein Family Expansions and Biological Complexity
Source: PLoS Comput Biol. 2006 May 26;2(5):e48. doi: 10.1371/journal.pcbi.0020048 (PMC1464810; doi:10.1371/journal.pcbi.0020048)
Supplement: Table S2 — Domains in the function categories are non-overlapping subsets of all domains in each organism. Only two function categories (i.e., domains of extracellular processes and regulation) show very good correlation of domain abundance and the number of different cell types (i.e. R ≥ 0.80). (110 KB DOC) Additional supporting material can be found at http://polaris.icmb.utexas.edu/people/cvogel/HV. The Web site contains several files: (1) mapping of the 50 more detailed function categories to the seven main function categories; and (2) names, SCOP identifiers, and SUPERFAMILY identifiers of all SCOP superfamilies, v. 1.69 [18]. The Web site also has links to additional Web pages, which display clusterings of superfamily expansion profiles using different cutoffs. Each of the Web pages describes clusters of similar expansion profiles, and the number and size of the clusters depends on the cutoffs used and the distribution of domain functions. Each cluster is labelled with a unique node number, and this number is taken directly from output of the XCluster program at http://genetics.stanford.edu/~sherlock/cluster.html. [file pcbi.0020048.st002.doc]

## Table S2. Only some groups of protein family expansions are correlated to the number of different cell types.

Domains in the function categories are non-overlapping subsets of the total number of domains in each organism. Only two function categories, i.e. domains of extra-cellular processes and regulation, show very good correlation of domain abundance and the number of different cell types, i.e. R≥0.80. R - Pearson correlation coefficient.

| Total | R | R^2^ |
| --- | --- | --- |
| Genes | 0.54 | 0.29 |
| Function |  |  |
| Extra-cellular processes | 0.96 | 0.91 |
| Regulation | 0.89 | 0.79 |
| Other | 0.76 | 0.58 |
| Intra-cellular processes | 0.74 | 0.54 |
| General | 0.61 | 0.38 |
| Information | 0.61 | 0.37 |
| Metabolism | 0.37 | 0.14 |
